# Age of blood and recipient factors determine the severity of transfusion-related acute lung injury (TRALI)

**DOI:** 10.1186/cc11178

**Published:** 2012-02-01

**Authors:** John-Paul Tung, John F Fraser, Maria Nataatmadja, Kathryn I Colebourne, Adrian G Barnett, Kristen M Glenister, Anna Y Zhou, Peter Wood, Christopher C Silliman, Yoke L Fung

**Affiliations:** 1 Research and Development, Australian Red Cross Blood Service, 44 Musk Avenue, Kelvin Grove, Brisbane, QLD 4059, Australia; 2 The School of Medicine, The University of Queensland, 288 Herston Road, Herston, Brisbane, QLD, Australia; 3 The Critical Care Research Group, The Prince Charles Hospital, Rode Road, Chermside, Brisbane, QLD 4059, Australia; 4 The Prince Charles Hospital, Rode Road, Chermside, Brisbane, QLD 4032, Australia; 5 Institute of Health and Biomedical Innovation and School of Public Health, Queensland University of Technology, 60 Musk Avenue, Kelvin Grove, Brisbane, QLD 4059, Australia; 6 Research and Development, Australian Red Cross Blood Service, Corner Balston and Kavanagh Streets, Southbank, Melbourne, VIC 3006, Australia; 7 Haematology Department, The Princess Alexandra Hospital, 199 Ipswich Road, Wooloongabba, Brisbane, QLD 4102, Australia; 8 Research Department, Bonfils Blood Center, 717 Yosemite Street, Denver, CO 80230, USA; 9 The Department of Pediatrics, School of Medicine, University of Colorado Denver, 12700 East 19th Avenue, Aurora, CO 80045, USA; 10 The Department of Surgery, School of Medicine, University of Colorado Denver, 12700 East 19th Avenue, Aurora, CO 80045, USA; 11 Cardiac Surgery Research Unit, The Prince Charles Hospital, Rode Road, Chermside, Brisbane, QLD 4059, Australia

## Abstract

**Introduction:**

Critical care patients frequently receive blood transfusions. Some reports show an association between aged or stored blood and increased morbidity and mortality, including the development of transfusion-related acute lung injury (TRALI). However, the existence of conflicting data endorses the need for research to either reject this association, or to confirm it and elucidate the underlying mechanisms.

**Methods:**

Twenty-eight sheep were randomised into two groups, receiving saline or lipopolysaccharide (LPS). Sheep were further randomised to also receive transfusion of pooled and heat-inactivated supernatant from fresh (Day 1) or stored (Day 42) non-leucoreduced human packed red blood cells (PRBC) or an infusion of saline. TRALI was defined by hypoxaemia during or within two hours of transfusion and histological evidence of pulmonary oedema. Regression modelling compared physiology between groups, and to a previous study, using stored platelet concentrates (PLT). Samples of the transfused blood products also underwent cytokine array and biochemical analyses, and their neutrophil priming ability was measured *in vitro*.

**Results:**

TRALI did not develop in sheep that first received saline-infusion. In contrast, 80% of sheep that first received LPS-infusion developed TRALI following transfusion with "stored PRBC." The decreased mean arterial pressure and cardiac output as well as increased central venous pressure and body temperature were more severe for TRALI induced by "stored PRBC" than by "stored PLT." Storage-related accumulation of several factors was demonstrated in both "stored PRBC" and "stored PLT", and was associated with increased *in vitro *neutrophil priming. Concentrations of several factors were higher in the "stored PRBC" than in the "stored PLT," however, there was no difference to neutrophil priming *in vitro*.

**Conclusions:**

In this *in vivo *ovine model, both recipient and blood product factors contributed to the development of TRALI. Sick (LPS infused) sheep rather than healthy (saline infused) sheep predominantly developed TRALI when transfused with supernatant from stored but not fresh PRBC. "Stored PRBC" induced a more severe injury than "stored PLT" and had a different storage lesion profile, suggesting that these outcomes may be associated with storage lesion factors unique to each blood product type. Therefore, the transfusion of fresh rather than stored PRBC may minimise the risk of TRALI.

## Introduction

There is currently heightened concern about the negative outcomes associated with transfusion of "older" or stored blood products. Several studies have identified the age of transfused packed red blood cell (PRBC) units as an independent risk factor for increased morbidity and mortality [1-4], including in the critical care setting [5,6]. The existence of contradictory studies [1,2,7,8], however, indicates that this is still a matter of conjecture which necessitates further research.

Transfusion-related acute lung injury (TRALI) is a serious and potentially fatal adverse transfusion event that has been associated with the transfusion of stored blood products [9-12]. Similar to acute lung injury (ALI) and acute respiratory distress syndrome (ARDS), TRALI manifests as respiratory distress with symptoms of hypoxaemia and pulmonary oedema [13-16]. However, in the case of TRALI, the onset of symptoms is temporally associated with transfusion (developing either during or within six hours of transfusion) [13-16]. TRALI has been reported by haemovigilance programs to be the most frequent cause of transfusion-related mortality in the US [17] and a leading cause of transfusion-related morbidity and mortality elsewhere [18,19]. However, TRALI is thought to be under-diagnosed and under-reported, particularly in the busy critical care setting where the development of symptoms may be attributed to multiple other disease processes or therapeutic interventions (for example, post cardiopulmonary bypass) rather than transfusion [20-22]. Indeed, a prospective study, which was not limited by the under-diagnosis and under-reporting inherent to haemo-vigilance programs, described the incidence of TRALI in the critical care setting as 8% [23], while a retrospective study described an incidence of 5% [24]. Interestingly, another prospective study reported an incidence of TRALI of 29% in end-stage liver disease (ESLD) patients admitted to critical care with gastrointestinal (GI) bleeding, suggesting that particular patient groups within the critical care setting may be at further risk of TRALI [25]. The normal rate of mortality in cases of TRALI is estimated to be 5 to 10% [16]; however, it may be higher in critical care patients as a mortality rate of 41% has been reported, although this was not adjusted for the influence of other morbidities [23].

TRALI is postulated to develop as the result of two separate clinical events [15,16]. The first or priming event is due to the patient's primary disease or condition which results in activation of the pulmonary endothelium and the accumulation of primed, adherent neutrophils in the lung [15,16]. The second event is the subsequent blood transfusion, whereby the primed neutrophils are activated by either a leucocyte antibody or biological response modifiers (BRM) present in the transfused blood product [15,16]. Activation of the primed neutrophils results in augmented release of their microbicidal arsenal, which causes collateral injury to the pulmonary endothelium that manifests as capillary leak, and clinically as TRALI [15,16]. Thus the two-event mechanism proposes that both recipient and blood product factors contribute to TRALI pathogenesis. Critical care patients may, therefore, be particularly susceptible to the development of TRALI, first, because of the severity of their underlying illness, and second, because they are more likely to receive blood transfusion [14,15,23,25].

Current risk reduction strategies (the preferential use of plasma from male donors, or the screening of donors for leucocyte antibodies) address the risk of TRALI associated with transfusion of leucocyte antibodies rather than BRM [17,26,27]. These BRM are thought to accumulate as part of the storage lesion of cellular blood products, such as packed red blood cell (PRBC) and platelet concentrates (PLT) [28-31]. Data from *in vivo *animal models as well as retrospective and *in vitro *studies indicate that stored PRBC or PLT may pose a greater risk of inducing TRALI than equivalent fresh PRBC or PLT [9-12]. The role of blood product factors, therefore, requires further elucidation. Using an established *in vivo *ovine model, this study investigated the hypotheses that: (i) both recipient factors (lipopolysaccharide (LPS) infusion to approximate clinical infection) and blood product factors (stored PRBC) would be required to induce TRALI, and (ii) that differences in the storage lesions of PRBC and PLT would result in differences in the haemodynamic and respiratory changes associated with the development of TRALI.

## Materials and methods

All experiments were approved by the University Animal Ethics Committee of the Queensland University of Technology, the Health Sciences Animal Ethics Committee of the University of Queensland and the Ethics Committee of the Australian Red Cross Blood Service, and conducted in accordance with the Australian Code of Practice for the Care and Use of Animals for Scientific Purposes.

### Collection and preparation of supernatant pools for transfusion

Seventy units of non-leucoreduced PRBC units were prepared by the Australian Red Cross Blood Service using standard procedures, including collection into citrate phosphate dextrose (CPD) and the addition of saline-adenine-glucose-mannitol (SAGM) additive solution. Thirty-five PRBC units were processed on Day 1 to obtain a "fresh PRBC" supernatant pool. The remaining 35 PRBC units were stored under standard conditions (4°C) until expiry (Day 42), when they were processed to obtain a "stored PRBC" supernatant pool. Supernatant pools were prepared by centrifugation as previously described [10] and were similarly heat-inactivated (56°C for 30 minutes) to eradicate the non-specific actions of complement and fibrinogen [12]. Similar pools of heat-inactivated Day 1 and Day 5 whole blood PLT supernatant were prepared in a previous study (d1-PLT-S/N or "fresh PLT" and d5-PLT-S/N or "stored PLT") [10], and aliquots were stored for further analyses in the present study.

### Transfusion protocol

The *in vivo *transfusion protocol has been previously described in detail [10]. Management of anaesthesia, mechanical ventilation, supplemental oxygen, voluemia and infusion/transfusion protocols were identical to the previous study [10]. Briefly, 28 female sheep (*Ovis aries*) received intravenous buprenorphine analgesia and ketamine/midazolam anaesthesia supplemented with butorphanol where required, and were mechanically ventilated and instrumented [10]. A one-hour period of stabilisation was allowed, after which hemodynamic monitoring and baseline bloods were collected. Sheep were randomly assigned into six groups to receive either saline or LPS as a first event, and then either saline or "fresh PRBC" or "stored PRBC" as a second event (Table 1). Either 30 ml of saline or LPS from *Escherichia coli *serotype O55:B5 (15 μg/kg based upon previous titration studies [10]; Sigma-Aldrich, Castle Hill, NSW, Australia) were infused intravenously into the sheep over 30 minutes (first event), followed by monitoring for 1 hour. For the second event, either saline or "fresh PRBC" or "stored PRBC" (10% of total blood volume) were infused into the sheep (200 ml/hr). Since the majority of clinical cases of TRALI develop within this time-frame [16], sheep were then monitored for two hours, after which they were euthanised with 12 ml pentobarbitone sodium (325 mg/ml; Virbac Animal Health, Milpera, NSW, Australia).

**Table 1:** Groups of sheep and incidence of TRALI

Group names	1^st ^event	2^nd ^event	n	Hypoxaemia	Pulmonary oedema	ALI/TRALI
						
						Number positive (% positive)
Sham	Saline	Saline	5	0	0	**0 (0%)**
Saline-fresh	Saline	"Fresh PRBC"	4	0	0	**0 (0%)**
Saline-stored	Saline	"Stored PRBC"	3	1	0	**0 (0%)**
LPS-control	LPS	Saline	6	0	2	**0 (0%)**
LPS-fresh	LPS	"Fresh PRBC"	5	1	1	**1 (20%)**
LPS-stored	LPS	"Stored PRBC"	5	5	4	**4 (80%)^*a*^**
Previous study [10]	LPS	"Fresh PLT" (d1-PLT-S/N)	5	2	3	**1 (20%)**

Previous study [10]	**LPS**	**"Stored PLT" (d5-PLT-S/N)**	**5**	**4**	**5**	**4 (80%)**

ALI, acute lung injury; d1-PLT-S/N, pooled heat-inactivated supernatant from Day 1 human platelet concentrates; d5-PLT-S/N, pooled heat-inactivated supernatant from Day 5 human platelet concentrates; "fresh PLT", pooled heat-inactivated supernatant from Day 1 human platelet concentrates; "fresh PRBC," pooled heat-inactivated supernatant from Day 1 human PRBC; LPS, lipopolysaccharide; n, number of sheep in group; "stored PLT", pooled heat-inactivated supernatant from Day 5 human platelet concentrates; "stored PRBC," pooled heat-inactivated supernatant from day 42 human packed red blood cell concentrates; TRALI, transfusion-related acute lung injury. Sheep were randomly assigned into the groups described above. Sheep were infused with either saline or LPS as a first event. Sheep were subsequently infused with saline, or transfused with either "fresh PRBC" or "stored PRBC" as a second event. TRALI was defined by the development of both hypoxaemia either during or within two hours of the second event transfusion, and pulmonary oedema upon blinded histological assessment of post- mortem lung sections. ^*a *^LPS infusion as a first event followed by the subsequent transfusion of stored PRBC as the second event predisposed the sheep towards the development of TRALI (by Fisher's exact test: *P *= 0.01 LPS-stored *vs*. saline-fresh, saline-stored and LPS-fresh without *post hoc *exclusion, or *P *= 0.048 LPS-stored *vs*. LPS-fresh and *P *= 0.003 LPS-stored *vs*. saline-fresh, saline-stored and LPS-fresh with *post hoc *exclusion).

### Sample collection

Samples of venous blood were collected at baseline, post first event infusion, post second event infusion, and pre-mortem. Samples of arterial blood for arterial blood gas (ABG) measurements were collected at 30-minute intervals throughout the experiment.

Post-mortem tissue samples were collected from the lower lobe of the left lung, for both histological and wet/dry weight analyses. Samples for histology were immediately fixed in 10% formalin and then processed and embedded in paraffin using routine methods. Histological examination of lung sections was as previously described [10]. In brief, 4 μm sections of lung underwent haemotoxylin and eosin staining before being semi-quantitatively assessed for pulmonary oedema by two researchers blinded to the experimental groupings. Twenty fields of lung histology for each section were photographed and graded for pulmonary oedema via a scoring system of 0- normal, 1- mild oedema, 2- moderate oedema and 3- severe oedema. Samples for wet/dry weight analysis were immediately weighed (wet weight) and then dried in an oven at 50°C until a stable weight was achieved (dry weight).

### Assessment of TRALI and ALI

TRALI was assessed as previously described by both the development of hypoxaemia during or within two hours of transfusion (second event) and histological evidence of pulmonary oedema (average score > 1) [10]. Hypoxaemia was defined as PaO_2_/FiO_2 _< 300 on at least two consecutive blood gas samples either during or following infusion of the second event. Where PaO_2_/FiO_2 _was below 300 prior to transfusion, a positive result for hypoxaemia was assessed by a worsening of PaO_2_/FiO_2 _for at least two consecutive blood gas samples either during or following transfusion. Sheep infused with saline as a control for transfusion were assessed for acute lung injury (ALI) rather than TRALI.

### Measurements and assays used

Physiological measurements were recorded continuously throughout the experiments as described previously [10]. Blood-gas analyses were performed on an automated blood gas analyser (ABL System 625, Radiometer, Copenhagen, Denmark).

Cytokine concentrations in the "fresh PRBC" and "stored PRBC" prepared in this study as well as the "fresh PLT" and "stored PLT" prepared previously [10], were semi-quantitatively characterised with a commercial microarray pre-loaded with 79 cytokines including epidermal growth factor (EGF), epithelial derived neutrophil activating 78 (ENA-78), growth related oncogene alpha (GRO), insulin-like growth factor-binding protein 1 (IGFBP-1), insulin-like growth factor (IGF), interleukin 8 (IL-8), interleukin 16 (IL-16), homologous to lymphotoxins, inducible expression, competes with HSV glycoprotein D for HVEM, a receptor expressed on T-lymphocytes (LIGHT), monocyte chemotactic protein 1 (MCP-1), macrophage inhibitory factor (MIF) and platelet-derived growth factor BB (PDGF-BB) (Human Cytokine Array V, RayBiotech, Atlanta, GA, USA). Analysis of the relative light intensity (RLI) of the corresponding spots via PDQuest Basic 2-D Gel Analysis Software (BioRad, Hercules, CA, USA) provided a relative measurement of the concentration of each specific cytokine or chemokine. Proteins that appeared to increase with storage were then quantified by commercial ELISA kits for EGF, ENA-78, GRO-α, IL-8, IL-16, and MCP-1 (R&D Systems, Minneapolis, MN, USA), and also for soluble CD40 ligand (sCD40L) (Bender MedSystems, Vienna, Austria) according to the manufacturers' instructions. Where concentrations were above the detection range of the kit, samples were diluted according to the manufacturers' instructions to obtain a value within the detectable range, which was then multiplied by the dilution factor to obtain the final concentration.

Using methods previously described [30-32], the ability of the PLT and PRBC supernatants to prime N-formylmethionyl-leucyl-phenylalanine (fMLP) induced respiratory burst function of human neutrophils was measured *in vitro *and compared to that of buffer, fresh autologous plasma and platelet-activating factor (PAF).

### Statistical analyses

The clinical incidence of TRALI was compared using two-way contingency tables and the Fisher's exact test. Physiological differences between groups were made using a two-way ANOVA with Bonferroni's multiple comparisons adjustment. Physiological data were subsequently modelled using a mixed model with a random intercept for each sheep in the R statistical package [33]. These models were used to examine differences between groups over time whilst accounting for repeated data [34]. Data from LPS-treated sheep that developed TRALI due to either "stored PRBC" or "stored PLT" transfusion were also compared using mixed modelling. Neutrophil priming ability of the different supernatants and controls was compared using a one-way ANOVA with Bonferroni's multiple comparisons adjustment. In all cases statistical significance was determined at *P *< 0.05.

## Results

### TRALI did not develop in healthy sheep

The absence of ALI in the sham group demonstrated that the anaesthetic, surgical and experimental protocols did not induce lung injury (Table 1). Similarly, sheep that were infused with saline as a control first event and then transfused with either "fresh PRBC" or "stored PRBC," (that is, saline-fresh and saline-stored groups) did not develop TRALI. A single sheep displayed evidence of hypoxaemia that worsened following transfusion of "stored PRBC."

### Transfusion of "stored PRBC" caused TRALI in LPS-primed sheep

**Figure 1:**
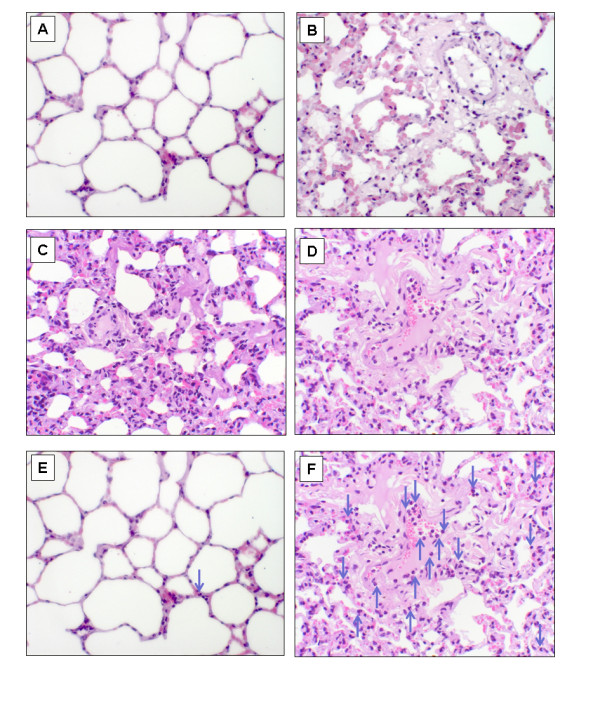
**Representative histology**. Representative haemotoxylin and eosin stained lung sections analysed histologically for pulmonary oedema. These range from no pulmonary oedema **(A) **through to mild **(B)**, moderate **(C) **and severe **(D) **pulmonary oedema. Neutrophils were identified by morphological assessment and are indicated by the blue arrows **(E **and **F)**. In contrast to sham sheep (E), there was widespread evidence of neutrophil infiltration in the lungs of LPS-stored sheep (F). LPS, lipopolysaccharide.

The dose of LPS, infused as a first event resulted in profound neutropenia (mean ± SD: 2.03 ± 0.86 neutrophils × 10^9^/L at baseline *vs*. 0.14 ± 0.03 neutrophils × 10^9^/L post-LPS; *P *< 0.0001 by paired t-test). The LPS-control group of sheep confirmed that this dose of LPS was insufficient to induce ALI (Table 1). One of the five LPS-fresh sheep and four of the five sheep in the LPS-stored group were diagnosed with TRALI (Table 1). This demonstrated that TRALI predominantly developed in sheep that received LPS-infusion followed by "stored PRBC" transfusion (*P *= 0.01 LPS-stored group *vs*. saline-fresh, saline-stored and LPS-fresh groups). Analysis of the lung section wet/dry weights (mean ± SD: sham = 5.33 ± 0.21; saline-fresh = 5.53 ± 0.37; saline-stored = 5.89 ± 0.52; LPS-control = 5.84 ± 0.96; LPS-fresh = 5.38 ± 0.73; LPS-stored = 6.99 ± 1.2) confirmed this finding (*P *= 0.0038 LPS-stored group *vs*. saline-fresh, saline-stored and LPS-fresh groups by unpaired t-test). There was evidence of widespread neutrophil infiltration in the lungs of LPS-stored sheep (Figure 1F) compared to those of sham sheep (Figure 1E). Of the six sheep that were diagnosed with hypoxaemia, five diagnoses (one in the LPS-fresh group and four in the LPS-stored group) were based upon a sustained worsening of hypoxaemia that was evident prior to transfusion of "stored PRBC."

### Haemodynamic and respiratory changes associated with the development of TRALI

**Figure 2:**
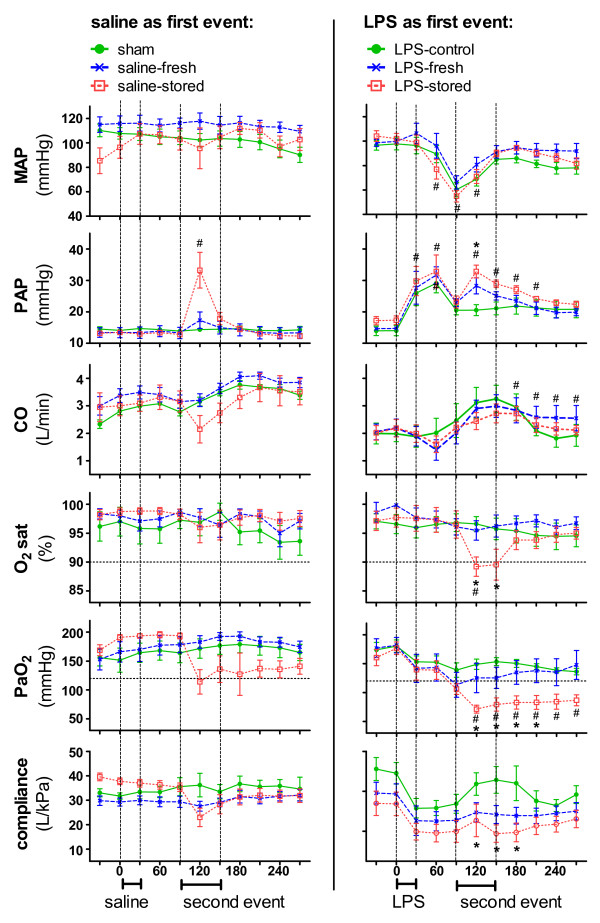
**Haemodynamic and respiratory changes**. Averaged data over 30-minute periods for each of the six groups of sheep. The first event (either saline or LPS) was infused from 0 to 30 minutes and the second event (either saline, "fresh PRBC" or "stored PRBC") was infused from 90 to 150 minutes. The left column represents sheep receiving saline-infusion as a first event (that is, sham, saline-fresh and saline-stored groups), and the right column represents sheep receiving LPS-infusion as a first event (that is, LPS-control, LPS-fresh and LPS-stored groups). Dashed lines at O_2_sat = 90% and PaO_2 _= 125 mmHg (FiO_2 _was 40%, therefore PaO_2_/FiO_2 _= 300) represent clinical cut-offs for hypoxaemia. LPS-stored sheep developed lower MAP, CO, PaO_2_, and O_2 _sat as well as higher PAP relative to sham sheep. Also, saline-stored sheep displayed increased PAP relative to sham sheep. # *P *< 0.05 *vs*. sham group using a two-way ANOVA with Bonferroni's multiple comparisons adjustment. LPS-stored sheep developed lower static pulmonary compliance, CO, PaO_2_, and O_2 _sat as well as higher PAP relative to the LPS-control group. * *P *< 0.05 *vs*. LPS-control using a two-way ANOVA with Bonferroni's multiple comparisons adjustment. ANOVA, analysis of variance; CO, continuous cardiac output; FiO_2_, fraction of inspired oxygen; "fresh PRBC," pooled heat-inactivated supernatant from Day 1 human PRBC; LPS, lipopolysaccharide; MAP, mean arterial pressure; O_2_sat, oxygen saturation; PaO_2_, arterial partial pressure of oxygen; PAP, pulmonary artery pressure; "stored PRBC," pooled heat-inactivated supernatant from Day 42 human PRBC; TRALI, transfusion-related acute lung injury.

Sheep in the LPS-stored group displayed changes to haemodynamic and respiratory changes relative to both sham and LPS-control groups' lower mean arterial pressure (MAP), cardiac output (CO), PaO_2_, and oxygen saturation (O_2 _sat) as well as higher pulmonary artery pressure (PAP) relative to sham (Figure 2; *P *< 0.05 for all comparisons). These sheep also displayed lower static pulmonary compliance (C_stat_), CO, PaO_2 _and O_2_sat (oxygen saturation) as well as higher PAP relative to LPS-control (*P *< 0.05 for all comparisons). There were also differences between the saline-stored and sham groups, demonstrated in an increased PAP relative to sham (Figure 2; *P *< 0.05), and while there was also a trend towards decreased PaO_2_, the increased PAP was the only significant change observed in the saline-stored and LPS-fresh groups.

To account for differences both in individual sheep and in groups of sheep, mixed modelling was used to further characterise the haemodynamic and respiratory changes associated with the development of TRALI (Table 2). Sheep in the LPS-stored group displayed changes relative to sham, LPS-control and LPS-fresh groups (Table 2; by mixed models: *P *< 0.001 for all comparisons).

**Table 2:** Haemodynamic changes

	Sham	Saline-fresh	Saline-stored	LPS-control	LPS-fresh	LPS-stored
	(*n *= 5)	(*n *= 4)	(*n *= 3)	(*n *= 6)	(*n *= 5)	(*n *= 5)
PAP (mmHg)	14.7 ± 0.7	18.4 ± 0.7	24.7 ± 0.7	27.0 ± 0.6	30.7 ± 0.7	37.0 ± 0.7^*a, b, c*^
	(10 to 19)	(4 to 28)	(9 to 60)	(5 to 47)	(6 to 62)	(13 to 89)
MAP (mmHg)	108.3 ± 2.2	107.3 ± 2.3	110.6 ± 2.3	90.0 ± 2.0	89.0 ± 2.3	98.1 ± 2.3^*a, c*^
	(67 to 146)	(64 to 172)	(55 to 155)	(37 to 130)	(44 to 169)	(30 to 149)
CVP (mmHg)	6.0 ± 0.6	5.1 ± 0.6	5.5 ± 0.6	5.7 ± 0.6	4.8 ± 0.6	5.2 ± 0.6^*a*^
	(1 to 20)	(0 to 13)	(0 to 20)	(0 to 17)	(0 to 18)	(1 to 18)
Heart rate (bpm)	102.5 ± 2.9	112.9 ± 3.0	113.7 ± 3.0	111.4 ± 2.7	121.8 ± 3.0	122.6 ± 3.0^a^
	(65 to 147)	(60 to 132)	(70 to 192)	(52 to 210)	(57 to 182)	(65 to 166)
O_2_sat (%)	97.2 ± 0.6	97.7 ± 0.7	96.8 ± 0.7	94.9 ± 0.6	95.4 ± 0.7	94.5 ± 0.7^*a*^
	(81 to 100)	(80 to 100)	(86 to 100)	(69 to 100)	(73 to 100)	(71 to 100)
EtCO_2 _(mmHg)	32.9 ± 1.2	32.5 ± 1.2	30.6 ± 1.2	34.0 ± 1.2	33.6 ± 1.2	31.7 ± 1.2
	(20 to 47)	(23 to 39)	(10 to 44)	(13 to 54)	(17 to 52)	(15 to 55)
CO (L/min)	4.6 ± 0.2	4.6 ± 0.2	3.7 ± 0.2	4.5 ± 0.2	4.5 ± 0.2	3.6 ± 0.2^*a, b, c*^
	(3.1 to 6.6)	(3.5 to 6.0)	(1.8 to 7.2)	(1.1 to 7.1)	(1.1 to 5.8)	(1.4 to 5.0)
SvO_2 _(%)	70.2 ± 1.5	73.3 ± 1.5	65.9 ± 1.5	72.5 ± 1.4	75.6 ± 1.5	68.3 ± 1.5^*a*^
	(44 to 82)	(33 to 81)	(38 to 84)	(48 to 91)	(48 to 86)	(22 to 77)
Body temperature (°C)	38.7 ± 0.1	38.5 ± 0.1	38.7 ± 0.1	38.7 ± 0.1	38.5 ± 0.1	38.7 ± 0.1
	(38.1 to 39.8)	(37.2 to 40.2)	(38.0 to 39.4)	(38.0 to 41.4)	(37.1 to 41.5)	(37.7 to 41.1)
PaO_2 _(mmHg)	184.8 ± 11.9	193.2 ± 13.0	152.6 ± 13.1	131.1 ± 7.4	139.5 ± 13.2	98.9 ± 13.2^*a, b, c*^
	(122 to 216)	(76.5 to 210)	(72.2 to 205)	(94.8 to 215)	(64.8 to 233)	(50.0 to 210)
PaCO_2 _(mmHg)	37.7 ± 2.0	37.0 ± 2.2	39.3 ± 2.2	39.8 ± 1.4	39.1 ± 2.1	41.4 ± 2.2^*a*^
	(30.5 to 42.3)	(24.9 to 40.6)	(22.7 to 51.5)	(28.5 to 54.5)	(23.7 to 51.9)	(27.7 to 70.6)
C_stat _(L/kPa)	38.4 ± 3.7	35.1 ± 4.0	30.4 ± 4.0	27.4 ± 2.5	24.1 ± 4.0	19.4 ± 4.0^*a, b, c*^
	(26.3 to 63.6)	(22.3 to 39.0)	(15.9 to 43.6)	(14.2 to 73.3)	(12.0 to 65.0)	(9.3 to 66.7)
PaO_2_/FiO_2 _(FiO_2 _= 40%)	462.0 ± 11.9	483.0 ± 13.0	381.5 ± 13.1	327.8 ± 7.4	348.8 ± 13.2	247.3 ± 13.2^*a, b, c*^
	(305 to 540)	(191 to 525)	(181 to 513)	(237 to 537)	(162 to 583)	(125 to 525)

CO, cardiac output; C_stat_, static pulmonary compliance; CVP, central venous pressure; EtCO_2_, end tidal carbon dioxide; FiO_2_, fraction of inspired oxygen; "fresh PRBC", pooled heat-inactivated supernatant from day 1 human packed red blood cell concentrates; LPS, lipopolysaccharide; LPS-control, group of sheep that received LPS followed by saline; LPS-fresh, group of sheep that received LPS followed by "fresh PRBC"; LPS-stored, group of sheep that received LPS followed by "stored PRBC"; MAP, mean arterial pressure; O_2_sat, oxygen saturation; PaCO_2_, arterial partial pressure of carbon dioxide; PaO_2_, arterial partial pressure of oxygen; saline-fresh, group of sheep that received saline followed by "fresh PRBC"; saline-stored, group of sheep that received saline followed by "stored PRBC"; PAP, pulmonary artery pressure; SEM, standard error of the mean; sham, group of sheep that received saline followed by saline; "stored PRBC", pooled heat-inactivated supernatant from day 42 human packed red blood cell concentrates; SvO_2_, mixed venous oxygen saturation; TRALI, transfusion-related acute lung injury.

Averages ± SEM (minimum-maximum) for each group are shown. Averages and SEM were calculated using mixed models. Minimum and maximum values were from recorded data. ^*a *^*P *< 0.05 *vs*. sham. ^*b *^*P *< 0.05 *vs*. LPS -control. ^*c *^*P *< 0.05 *vs*. LPS-fresh.

### "Stored PRBC" caused more severe injury than "stored PLT"

To investigate whether TRALI induced by "stored PRBC" was more severe than that induced by "stored PLT" in a previous study [10], data from the four sheep that developed TRALI in each of these two groups were used to create new mixed models (Table 3). These analyses indicated that TRALI induced by "stored PRBC" resulted in lower MAP (*P *< 0.0001) and CO (*P *= 0.0145), as well as higher CVP (*P *< 0.0001) and body temperature (*P *< 0.0001) relative to that induced by "stored PLT". There were also trends towards lower PaO_2 _and increased PAP, although these did not reach significance in this study. These changes indicate that TRALI induced by "stored PRBC" results in more severe haemodynamic changes than that induced by "stored PLT".

**Table 3:** Comparison of haemodynamic changes in TRALI induced by either "stored PLT" or "stored PRBC"

	"stored PLT" (*n *= 4)	"stored PRBC" (*n *= 4)	*P*
PAP (mmHg)	24.4 ± 2.1	28.7 ± 2.4	0.0946
MAP (mmHg)	87.9 ± 5.5	74.5 ± 5.6	< 0.0001
CVP (mmHg)	3.0 ± 1.2	7.8 ± 1.2	< 0.0001
Heart rate (bpm)	112.7 ± 4.7	117.3 ± 5.4	0.5262
O_2_sat (%)	92.6 ± 2.8	89.5 ± 3.1	0.3598
EtCO_2 _(mmHg)	38.8 ± 3.0	33.3 ± 5.6	0.2253
CO (L/min)	4.9 ± 0.3	3.7 ± 0.4	< 0.05
SvO_2 _(%)	70.6 ± 5.9	62.3 ± 6.8	0.3499
Body temp (°C)	39.1. ± 0.3	40.3 ± 0.3	< 0.0001
PaO_2 _(mmHg)	103.9 ± 14.3	68.5 ± 16.4	0.1086
PaCO_2 _(mmHg)	42.7 ± 3.3	41.0 ± 3.7	0.7228
C_stat _(L/kPa)	17.5 ± 3.0	15.7 ± 2.4	0.6305
PaO_2_/FiO_2_	259.8 ± 14.3	171.3 ± 16.4	0.1086

CO, cardiac output; C_stat_, static pulmonary compliance; CVP, central venous pressure; EtCO_2_, end tidal carbon dioxide; n, number of sheep in group; FiO_2_, fraction of inspired oxygen (= 40%); LPS, lipopolysaccharide; MAP, mean arterial pressure; O_2_sat, oxygen saturation; PaCO_2_, arterial partial pressure of carbon dioxide; PaO_2_, arterial partial pressure of oxygen; PAP, pulmonary artery pressure; "stored PLT", pooled heat-inactivated supernatant from Day 5 non-leucoreduced human platelet concentrates; "stored PRBC", pooled heat-inactivated supernatant from Day 42 non-leucoreduced human packed red blood cell concentrates; SEM, standard error of the mean; SvO_2_, mixed venous oxygen saturation; TRALI, transfusion-related acute lung injury. Averages ± SEM for each group are shown. Data and *P*-values were calculated using mixed modelling.

### Differences in the composition of these products may explain the differing injury observed

We hypothesised that the observed haemodynamic differences may be related to differences in the composition of the different blood products. Biochemical changes related to storage in the "stored PRBC" and "stored PLT" were normal for stored blood products (Table 4).

**Table 4:** Supernatant composition

	PRBC		PLT	
	"fresh PRBC"	"stored PRBC"	"fresh PLT"	"stored PLT"
Hb (g/dL)	0.01	0.33	0.01	0.02
K^+ ^(mmol/L)	2.0	44.4	3.9	4.4
Na^+ ^(mmol/L)	143	105	157	158
Cl^- ^(mmol/L)	112	103	74	77
Glucose (mmol/L)	28.0	14.3	7.0	0.0
Lactate (mmol/L)	6.5	29.6	4.6	18.0
sCD40L (ng/ml)	0.24	1.50	0.48	0.73
EGF (pg/ml)	0	298	5	69
ENA-78 (pg/ml)	63	1,555	226	6,333
GRO-α (pg/ml)	352	662	110.1	692
IL-8 (pg/ml)	28	10,004	323	379
IL-16 (pg/ml)	50	19,754	225	260
MCP-1 (pg/ml)	0	422	0	0

Cl^-^, chlorine anion; EGF, epidermal growth factor; ENA-78, epithelial derived neutrophil activating 78; "fresh PLT", pooled heat-inactivated supernatant from Day 1 non-leucoreduced human platelet concentrates; "fresh PRBC", pooled heat-inactivated supernatant from Day 1 non-leucoreduced human packed red blood cell concentrates; GRO-α, growth related oncogene alpha; Hb, haemoglobin; IL-8, interleukin 8; IL-16, interleukin 16; K^+^, potassium cation; MCP-1, monocyte chemotactic protein 1; Na^+^, sodium anion; PLT; platelet concentrates; PRBC; packed red blood cell concentrates; sCD40L, soluble CD40 ligand; "stored PLT", pooled heat-inactivated supernatant from Day 5 non-leucoreduced human platelet concentrates; "stored PRBC", pooled heat-inactivated supernatant from Day 42 non-leucoreduced human packed red blood cell concentrates. Both "stored PRBC" and "stored PLT" demonstrated increased levels of lactate and sCD40L as well as decreased pH and glucose levels compared to fresh equivalents. Additionally "stored PRBC" demonstrated increased haemoglobin and potassium levels compared to "fresh PRBC" and to "stored PLT". Higher concentrations of EGF, ENA-78 and GRO-α, were present in both "stored PLT" and "stored PRBC" relative to fresh equivalents. However the concentrations of IL-8, IL-16 and MCP-1 were only increased only "stored PRBC". Additionally, concentrations of EGF, IL-8, IL-16 and MCP-1 were all higher in "stored PRBC" than in "stored PLT". Conversely, concentrations of ENA-78 and GRO-α were higher in "stored PLT" than in "stored PRBC".

Soluble CD40L levels were measured because this molecule has been associated with TRALI pathogenesis [29,35]. Storage resulted in accumulation of sCD40L in both "stored PRBC" and in "stored PLT," with the former having higher levels (Table 4). However, these levels were much lower than those previously reported in units of Day 42 PRBC or Day 5 PLT [29,35]. To investigate whether heat-inactivation may also have reduced levels of sCD40L, we measured levels in equivalent non-heat-inactivated supernatant pools: PLT (Day 1: 1.85 ng/ml *vs*. Day 5: 9.25 ng/ml) and PRBC (Day 1: 0.40 ng/ml *vs*. Day 42: 8.36 ng/ml). This confirmed that heat-inactivation was responsible for the reduced levels of sCD40L evident in both "stored PRBC" and "stored PLT."

In contrast to sCD40L, no significant differences resulting from heat-inactivation were found in the concentrations of EGF, ENA-78, GRO-α, IL-8, IL-16 and MCP-1 (data not shown). As shown in Table 4 the concentrations of the EGF, ENA-78 and GRO-α, were increased in both the "stored PRBC" and the "stored PLT" compared to the respective fresh product ("fresh PRBC" and "fresh PLT"). However, concentrations of IL-8, IL-16 and MCP-1 were only increased in "stored PRBC" (Table 4). Hence, there were more storage-related changes in cytokine concentration in "stored PRBC" than in "stored PLT." Comparison of the two stored products also revealed that "stored PRBC" contained higher concentrations of EGF, IL-8, IL-16 and MCP-1, while "stored PLT" contained higher concentrations of ENA-78 (Table 4). Together these results demonstrate that there were storage-related changes, which were different depending on the blood product type (that is, "stored PRBC" *vs*. "stored PLT").

### Differing composition did not result in differences in neutrophil priming

**Figure 3:**
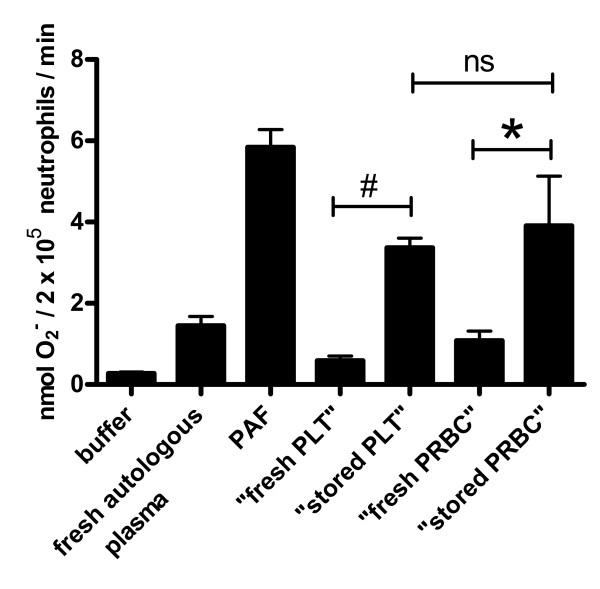
**Neutrophil priming ability**. Ability to prime fMLP-induced human neutrophil respiratory burst function is shown as mean of *n *= 4 experiments. Error bars indicate SEM. Both "stored PRBC" and "stored PLT" display increased ability to prime fMLP-induced neutrophil respiratory burst than the equivalent fresh product. There was no difference between the ability of "stored PRBC" and "stored PLT" to prime fMLP-induced neutrophil respiratory burst. # *P *< 0.001 "stored PLT" *vs*. "fresh PLT" using a one-way ANOVA with Bonferroni's multiple comparisons adjustment. * *P *< 0.001 "stored PRBC" *vs*. "fresh PRBC" using a one-way ANOVA with Bonferroni's multiple comparisons adjustment. ns *P *> 0.05 "stored PRBC" *vs*. "stored PLT" using a one-way ANOVA with Bonferroni's multiple comparisons adjustment. ANOVA, analysis of variance; fMLP, N-formylmethionyl-leucyl-phenylalanine; "fresh PLT", pooled heat-inactivated supernatant from Day 1 non-leucoreduced human platelet concentrates; "fresh PRBC", pooled heat-inactivated supernatant from Day 1 non-leucoreduced human packed red blood cell concentrates; min, minute; O_2_^-^, superoxide anion; PAF, platelet activating factor; PLT, platelet concentrate, PRBC packed red blood cells; "stored PLT", pooled heat-inactivated supernatant from Day 5 non-leucoreduced human platelet concentrates; "stored PRBC", pooled heat-inactivated supernatant from day 42 non-leucoreduced human packed red blood cell concentrates; SEM, standard error of the mean.

Because the neutrophil respiratory burst function plays a key role in the pathogenesis of TRALI [36], we compared the ability of "stored PRBC" and "stored PLT" to prime fMLP-induced activation of the respiratory burst in human neutrophils *in vitro*. In accordance with previous studies [31,37], "stored PRBC" and "stored PLT" both demonstrated greater priming ability than equivalent fresh supernatants (*P *< 0.001 in both cases; Figure 3). However, no difference was observed between the "stored PRBC" and the "stored PLT" (Figure 3).

## Discussion

The development of respiratory dysfunction compromises the recovery of severely ill patients and may contribute to their morbidity and death. While some patients may progress to either ALI or ARDS, the association with recent blood transfusion may be overlooked [20-22]. Thus, many cases of ALI/ARDS may in fact represent TRALI, and the true scale of the risks posed by TRALI in the critical care setting are likely to be under-appreciated. Prospective studies have revealed an incidence of TRALI ranging from 5 to 8% in general critical care patients [23,24] and up to 29% in ELSD critical care patients admitted with GI bleeding [25]. This study provides additional evidence that both patient and blood product factors contribute to the development of TRALI, and that the type of blood product influences the severity of injury.

Patients with severe illness are hypothesised to be more likely to develop TRALI, thus critically ill patients may be particularly susceptible to the development of TRALI [14,22]. In this study, TRALI only developed in "ill" sheep and did not develop in any of the "healthy" sheep, even following transfusion of "stored PRBC." This is consistent with previous TRALI models, in which both a clinical first event, either LPS-infusion or, in the case of mice, their exposure to a germ environment, and an appropriate second event (that is, stored blood or leucocyte antibody) were required for TRALI to develop [9,10,12,38,39]. Thus, this study reaffirms the importance of patient factors in the development of TRALI.

The age of the transfused blood product was also found to be crucial to the development of TRALI, as it predominantly developed in LPS-primed sheep transfused with "stored PRBC" and not "fresh PRBC." This adds to findings from previous *in vivo *models in which TRALI has been described subsequent to transfusion with supernatant from stored human PRBC in rats [12] or stored human PLT in both rats [40] and in sheep [10]. During routine storage of PRBC and PLT, proteins and lipids (or their metabolites) are released by cells into the storage medium [28-31,41,42]. These soluble factors are retained in the supernatant and are thought to contribute to the development of TRALI [1-3,5,6,11,12,28-31,43-45], although some studies have also implicated the transfused cells [44,46]. In this study, cytokine array and ELISA analyses were used to identify the soluble factors that may have contributed to the development of TRALI in the sheep. It was demonstrated that "stored PRBC" contained higher levels of EGF, ENA-78, GRO-α, IL-8, IL-16 and MCP-1 relative to "fresh PRBC", while levels of lactate and potassium increased and levels of sodium decreased. Since neutrophils are key effector cells in TRALI pathogenesis, the biological relevance of these changes was confirmed by the increased *in vitro *neutrophil priming ability present in "stored PRBC" compared to "fresh PRBC".

Heat-inactivation of the human blood product supernatant used in this and in previous studies [9,10,12,28,39] was necessary to prevent widespread thrombus formation and mortality due to non-specific actions of complement and fibrinogen [9,12,39]; however, represents a limitation of these models. As was demonstrated for sCD40L, heat-inactivation may reduce the concentration of some protein BRMs; however, levels of EGF, ENA-78, GRO-α, IL-8, IL-16 and MCP-1 were all unaffected by heat-inactivation. It remains possible that heat-inactivation may have affected other parameters not investigated, and may have influenced the development of TRALI. The alternative approach of transfusing homologous ovine with PRBC rather than with heat-inactivated supernatant from human PRBC was not used in this study because of the limitations of this alternative approach. First, while the preparation of ovine PRBC is not technically difficult, this process requires standardisation and validation to ensure that the ovine PRBC provide a suitable model of human PRBC. Second, as has been demonstrated in small animal models [45,47-49], there are likely to be differences between the storage lesions of ovine and human PRBC. Detailed comparative data comparing the storage lesions of ovine PRBC and human PRBC are, therefore, essential to validate an ovine model of homologous transfusion for the study of effects related to the age of blood. While future studies are planned to address these limitations of homologous transfusion models, it was felt that, at the present time, the transfusion of heat-inactivated supernatant from human blood products, provided a more relevant clinical model of TRALI.

PRBC that have not undergone pre-storage leucoreduction comprise a significant proportion of the PRBC used in the USA (approximately 20% of the approximately 17 million PRBC transfused in 2009) [50]. Hence, the findings of this study are of particular clinical relevance to the USA and other countries in which universal pre-storage leucoreduction of blood products has not yet been implemented. Leucoreduction has been shown to reduce the concentration of leucocyte-derived factors in the storage lesion of cellular blood products [41]; however, whether it also reduces the risk of TRALI remains a matter of conjecture based upon current evidence [7,12,18,51]. Of note, analyses of 89 TRALI cases from two tertiary care medical centres in the USA [7] and of 60 TRALI cases in The Netherlands [8] failed to demonstrate any association between the length of storage of leucoreduced PRBC and TRALI, although these analyses may have been confounded by the presence of leucocyte antibodies in a proportion of leucoreduced PRBC. Hence, the importance of the present study, and the ovine model, as a historical marker allowing for further investigation of the effects of leucoreduction upon TRALI pathogenesis. Accordingly, follow-up studies using equivalent leucoreduced PRBC have been planned to identify common BRM and to elucidate the effects that transfusion of supernatant from stored leucoreduced human PRBC may have upon TRALI pathogenesis in the ovine model. These effects may then be compared to those reported in the present study.

The definition of TRALI used in this study included cases in which a sustained worsening of pre-existing hypoxaemia was evident following transfusion. This was justified because the control groups and detailed monitoring used in the experimental setting make it possible to clearly define such cases. Two separate analyses confirmed the robustness of these data. First, analyses of averaged data demonstrated that the LPS-stored group had lower PaO_2 _values post-transfusion compared to the LPS-control group (Figure 2). Second, analyses using mixed modelling demonstrated that the LPS-stored group had lower PaO_2 _values post-transfusion compared to both the LPS-control and LPS-fresh groups (Table 2). Thus, it was possible to conclude that the worsening hypoxaemia was related to the transfusion of "stored PRBC" rather than the continued effects of LPS-infusion. In contrast to the experimental setting, defining worsening hypoxaemia related to transfusion is problematic in the clinical setting. Therefore, more restrictive criteria, in which TRALI is only defined by the onset of new hypoxaemia, are used clinically [52,53]. However, Koch *et al*. have demonstrated that, regardless of transfusion history, over 60% of cardiac surgical patients were hypoxaemic upon admission to ICU, this highlighting the difficulty in applying current TRALI definitions in the critical care setting [54].

The relative similarity in pulmonary anatomy and physiology between sheep and humans [55-59] represents a significant advantage of this ovine model over existing small animal rodent models of TRALI. Another distinct advantage is the larger size of the sheep relative to the rats and mice used for other TRALI models. This enabled detailed monitoring of the respiratory and haemodynamic changes associated with TRALI. In sheep that developed TRALI, the observed reduction in C_stat _and decrement in oxygenation represents a physiological manifestation of the loss of the open alveolar structure evident upon post-mortem histological analysis. The continuous physiological monitoring also revealed that TRALI was associated with development of pulmonary arterial hypertension, further increasing the workload on the right heart, which may lead to poorer tissue oxygenation, with increased venous pressures and reduced cardiac output. Hence, TRALI worsens oxygenation at the arterio-alveolar interphase, as well as diminishing tissue oxygen delivery, due to the cardiovascular perturbations. In addition to predisposing the development of TRALI, it is possible that by worsening tissue dysoxia in other organs the transfusion of stored blood might also contribute to the development of multiple organ dysfunction syndrome (MODS) [21,60], although further studies would be required to investigate this hypothesis.

This study provides further evidence that both recipient (first event) and blood product (second event) factors contribute to the development of TRALI. Such a two-event mechanism was first postulated for some instances of ARDS [61], then was adapted for TRALI [15], and recently has been re-stated as a threshold mechanism for TRALI [14]. This proposes that the development of TRALI is associated with both the severity of underlying illness and the strength of blood product factors [14]. This interaction may provide an explanation for both the unexpected lack of TRALI in a single LPS-infused sheep transfused with "stored PRBC" as well as the unexpected development of TRALI in a single LPS-infused sheep transfused with "fresh PRBC." In the former case, it is possible that recipient factors were protective against TRALI. Genetic factors have been implicated in the development of ALI [62], and it is possible that they may also play a role in TRALI as only some patients transfused with stored PRBC go on to develop TRALI. In the latter case, *post hoc *analyses revealed that abnormal baseline respiratory data were indicative of pre-existing lung injury (initial PaO_2_/FiO_2 _was 277.5, which recovered to 452.5 at the start of the experiment). Therefore, we speculate that pre-existing injury in combination with LPS-infusion may have rendered this sheep more susceptible to the development of TRALI, such that a weaker second event stimulus ("fresh PRBC") was sufficient to induce TRALI. This would be consistent with the proposed threshold mechanism. Thus, critical care patients may be particularly susceptible to the development of TRALI because of the severity of their illness. In addition, the risk of developing TRALI may be further increased if they are transfused with stored blood products which have a higher BRM content [1-3,5,6,11,12,28-31,43-45].

Finally, this study demonstrated that the injury profile of TRALI induced by "stored PRBC" was more severe than that previously described by "stored PLT" [10]. Data re-modelling confirmed a reduction in MAP and CO as well as higher CVP and temperature in TRALI induced by "stored PRBC." The strength of the recipient factors was consistent, as the same dose of LPS was used in both studies [10]. Therefore, the difference in symptoms may be attributable to a difference in blood product factors. This is supported by the higher concentrations of EGF, IL-8, IL-16, MCP-1, lactate and potassium measured in "stored PRBC" than in "stored PLT." The observation that these higher concentrations, present in the transfused blood product were associated with more severe symptoms is suggestive of a dose-response relationship; however, further research would be required to confirm this hypothesis. Also, the mechanism by which each of these potential BRM may act requires further elucidation. As no differences were observed in the *in vitro *neutrophil priming ability of "stored PRBC" and "stored PLT," direct actions of these potential BRMs upon neutrophils are unlikely to contribute to the differences observed *in vivo*. It is possible that other cells, such as platelets [38], T-lymphocytes [63] and endothelial cells [16], which also contribute to the pathophysiology of TRALI, may have contributed to the observed haemodynamic differences and this warrants further investigation.

## Conclusions

This study has confirmed that the transfusion of soluble factors present in stored blood products (that is, PRBC) presents a significantly increased risk of TRALI compared to equivalent fresh products. Symptoms associated with TRALI induced by "stored PRBC" were more severe than for TRALI induced by "stored PLT," possibly due to an increased range and concentration of cytokines and other factors present in the "stored PRBC," and is suggestive of a dose-response relationship. Improved understanding of the injurious soluble factors present in stored blood products is required to direct the manufacture of safer blood components. This study has also reaffirmed the importance of recipient (patient) factors in the development of TRALI as only LPS-primed sheep went on to develop TRALI. Hence, severely ill patients, such as those in critical care units, may be at increased risk of developing TRALI. This study reaffirms the fact that blood transfusion has associated risk and should be prescribed with prudence, particularly in the critical care setting.

## Key messages

• In an *in vivo *ovine model, sick sheep (that is, infused with LPS: 15 μg/kg *i.v*.) transfused with "stored PRBC" predominantly developed TRALI (*P *= 0.01 *vs*. control groups).

• Development of TRALI induced by "stored PRBC" was associated with haemodynamic changes that were more severe than in TRALI induced by "stored PLT" in a previous study [10], and may be because of differences in the storage lesions of "stored PRBC" and "stored PLT."

• The age of the blood product transfused (that is, fresh *vs*. date-of-expiry), the type of blood product transfused (that is, PRBC *vs*. WB-PLTs) and the health of the recipient (that is, saline *vs*. LPS as a first event), therefore, all contributed to determine the severity of TRALI in the ovine model.

• Critical care patients may, therefore, be particularly susceptible to development of TRALI because of the severity of their illness and their increased reliance upon transfusion of blood products, and this susceptibility may be further increased with the transfusion of stored blood products.

## Abbreviations

ABG: arterial blood gas; ALI: acute lung injury; ARDS: acute respiratory distress syndrome; BRM: biological response modifier; CO: cardiac output; CPD: citrate phosphate dextrose; C_stat_: static pulmonary compliance; CVP: central venous pressure; d1-PLT-S/N: pooled heat-inactivated supernatant from Day 1 non-leucoreduced human whole blood platelet concentrates; d5-PLT-S/N: pooled heat-inactivated supernatant from Day 5 non-leucoreduced human whole blood platelet concentrates; EGF: epidermal growth factor; ENA-78: epithelial derived neutrophil activating 78; EtCO_2_: end tidal carbon dioxide; ESLD: end-stage liver disease; FFP: fresh frozen plasma; FiO_2_: fraction of inspired oxygen; fMLP: N-formylmethionyl-leucyl-phenylalanine; "fresh PLT": pooled heat-inactivated supernatant from Day 1 non-leucoreduced human platelet concentrates; "fresh PRBC": pooled heat-inactivated supernatant from Day 1 non-leucoreduced human packed red blood cell concentrates; GI: gastrointestinal; GRO-α: growth related oncogene alpha; HETE: hydroxyl eicosotetranoic acid; IGFBP-1: insulin-like growth factor-binding protein 1; IL-8: interleukin 8; IL-16: interleukin 16; LPS: lipopolysaccharide; MAP: mean arterial pressure; MCP-1: monocyte chemotactic protein 1; MIF: macrophage inhibitory factor; MODS: multiple organ dysfunction syndrome; N/A: not applicable; O_2_^-^: superoxide anion; O_2_sat: oxygen saturation; PaCO_2_: arterial partial pressure of carbon dioxide; PAF: platelet activating factor; PaO_2_: arterial partial pressure of oxygen; PAP: pulmonary artery pressure; PDGF-BB: platelet-derived growth factor BB; PLT: platelet concentrate; PRBC: packed red blood cells; RLI: relative light intensity; SAGM: saline-adenine-glucose-mannitol; sCD40L: soluble CD40 ligand; "stored PLT": pooled heat-inactivated supernatant from Day 5 non-leucoreduced human platelet concentrates; "stored PRBC": pooled heat-inactivated supernatant from Day 42 non-leucoreduced human packed red blood cell concentrates; SvO_2_: mixed venous oxygen saturation; TRALI: transfusion-related acute lung injury.

## Competing interests

JPT, KIC, KMG, AYZ and YLF were employees of the Australian Red Cross Blood Service during the completion of this study. The authors declare that they have no other competing interests.

## Authors' contributions

JPT, JFF, KIC, AGB, PW, CSS and YLF contributed to obtaining funding for this study and the conception and design of the study. JPT, KIC and YLF co-ordinated the provision of PRBC, and contributed to the supernatant preparation and pooling. JPT, JFF, KIC and YLF contributed to the animal experiments. JPT, MN, KMG, AYZ and YLF contributed to the laboratory testing. All authors contributed to the analysis and interpretation of data as well as to the preparation of this manuscript.
